# Nec-1 Attenuates Neurotoxicity Induced by Titanium Dioxide Nanomaterials on Sh-Sy5y Cells Through RIP1

**DOI:** 10.1186/s11671-020-03300-5

**Published:** 2020-03-27

**Authors:** Ting Zhou, Wei-kun Huang, Qiu-yan Xu, Xue Zhou, Yue Wang, Zhao-hui Yue, Bin Song

**Affiliations:** Guizhou Provincial People’s Hospital, Guiyang, 550002 China

**Keywords:** Titanium dioxide nanoparticles, Neurotoxicity, Inflammatory injury, Necroptosis, Nec-1, Rip1

## Abstract

Titanium dioxide nanomaterials are applied in numerous fields due to their splendid physicochemical characteristics, which in turn poses a potential threat to human health. Recently, numerous in vivo studies have revealed that titanium dioxide nanoparticles (TNPs) can be transported into animal brains after exposure through various routes. Absorbed TNPs can accumulate in the brain and may disturb neuronal cells, leading to brain dysfunction. In vitro studies verified the neurotoxicity of TNPs. The mechanisms underlying the neurotoxicity of TNPs remains unclear. Whether necroptosis is involved in the neurotoxicity of TNPs is unknown. Therefore, we performed an in vitro study and found that TNPs induced inflammatory injury in SH-SY5Y cells in a dose-dependent way, which was mitigated by necrostatin-1 (Nec-1) pretreatment. Since receptor-interacting protein kinase 1 (RIP1) is reported to be the target of Nec-1, we silenced it by siRNA. We exposed mutant and wild-type cells to TNPs and assessed inflammatory injury. Silencing RIP1 expression inhibited inflammatory injury induced by TNPs exposure. Taken together, Nec-1 ameliorates the neurotoxicity of TNPs through RIP1. However, more studies should be performed to comprehensively assess the correlation between the neurotoxicity of TNPs and RIP1.

## Introduction

Owing to their splendid physicochemical properties, titanium dioxide nanomaterials are synthesized [1] and widely used for various purposes, such as cosmetics [2], industry fields [3, 4], and medical areas [5]. However, this widespread usage may pose a big threat to human health [6]. Once exposed, the majority of titanium dioxide nanoparticles (TNPs) enters the human body through inhalation and ingestion [6]. The respiratory system [7], digestive system [8], and cardiovascular system [9] all may be interrupted by absorbed TNPs. Similarly, the brain, as the most important part of the central nervous system, can also be disturbed, since TNPs in the circulation can penetrate the blood-brain barrier, and inhaled NPs could be transported into the brain through the olfactory pathway [10]. Once TNPs enter the brain, they may accumulate there and cause damage to the brain, leading to dysfunctions. Damage to the brain is usually irreversible and severe, and therefore any potential cause of damage should be investigated [11].

Although no epidemiologic study has explored the association between TNPs exposure and brain diseases, plenty of in vivo and in vitro studies have confirmed the neurotoxicity of TNPs [12]. Moreover, the major focus has been on research to uncover the underlying mechanisms. For this purpose, we previously reviewed the currently known molecular mechanisms of TNPs neurotoxicity and found that programmed cell death (PCD) processes, such as apoptosis and autophagy, were implicated in the neurotoxicity of TNPs [13].

Necroptosis, also called regulated necrosis, is another type of PCD. Unlike apoptosis which is caspase-dependent, necroptosis is a receptor-interacting protein kinase 1/3 (RIP1/RIP3)-dependent. Activated RIP1 recruits RIP3 to form a necrosome, which activates mixed lineage kinase domain-like protein (MLKL) to initiate necroptosis [14]. Necroptosis can regulate cell death and neuroinflammation [15, 16], which are both involved in the neurotoxicity of TNPs. Therefore, we hypothesized that necroptosis is implicated in the neurotoxicity caused by TNPs.

To test our hypothesis, we performed an in vitro study to explore the role of necroptosis in the neurotoxicity of TNPs. In this study, cell viability, LDH leakage, and inflammatory cytokines (TNF-α, IL-1β, IL-6, and IL-8) were measured after TNPs exposure. First, SH-SY5Y cells were cultured with various concentrations of TNPs. Second, cells were exposed to TNPs with or without Nec-1, which is a potent inhibitor of necroptosis. Third, RIP1 gene expression, which can be suppressed by Nec-1, was silenced using siRNA, after which mutant and wild-type cells were treated with TNPs. This study sheds light on the comprehensive understanding of the molecular mechanisms underlying neurotoxicity of TNPs.

## Results

### TNPs Inhibit Cell Viability

**Fig. 1:**
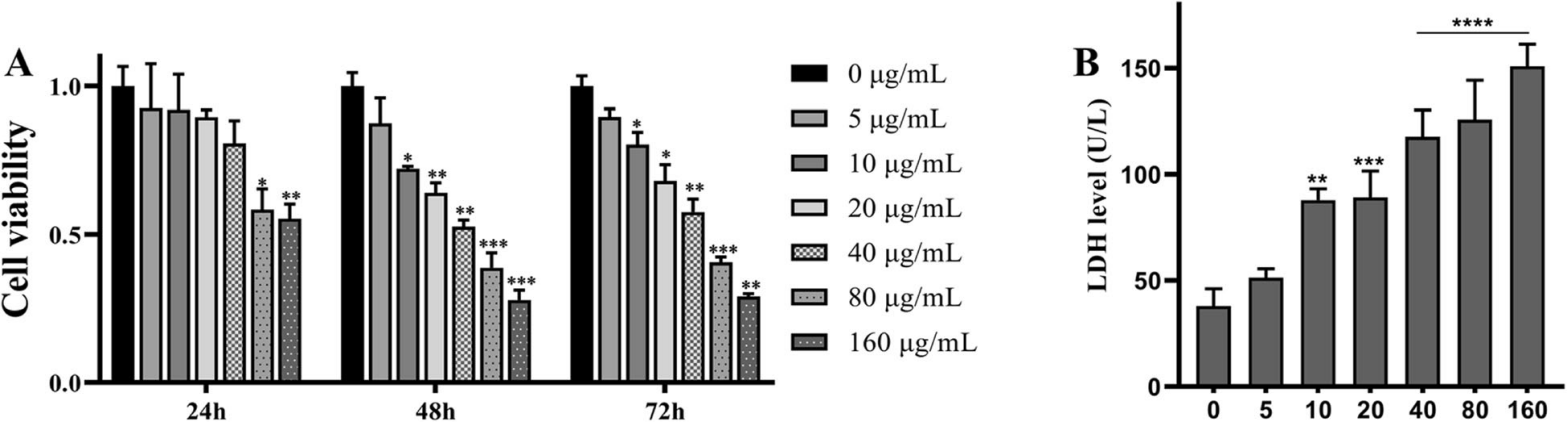
Different concentrations of TiO2-NPs exposure on cell viability of SH-SY5Y cells at 24, 48, and 72 h and LDH leakage at 72 h. (Compared with the control group, ^*^p < 0.05, ^**^p < 0.01, ^***^p < 0.001, ^****^p < 0.0001)

To verify the cytotoxicity of TNPs on SH-SY5Y cells, we first assessed cell viability using CCK8 assay. Cells were cultured with TNPs concentrations ranging from 5 to 160 μg/mL for 24, 48, and 72 h. As shown in Fig. 1, cell viability remained unchanged in the cells treated with 5, 10, 20, and 40 μg/mL after 24 h of exposure. When cells were treated for 48 and 72 h, cell viability remained unchanged only in the 5 μg/mL group (*p* = 0.4507 at 48 h and *p* = 0.1002 at 72 h). Moreover, cell viability was dramatically lower in the cells treated with 80 μg/mL TNPs after 48 (*p* = 0.0007) and 72 h (*p* = 0.0008). Based on those results, we concluded that TNPs decreased cell viability in a dose- and time-dependent way (data not shown), indicating that long-term exposure was more toxic.

### TNPs Damage Membrane Integrity

Nest, we analyzed the toxic effects of TNPs on membrane integrity after 72 h of exposure. Membrane integrity was assessed by measuring LDH leakage. As shown in Fig. 1b, LDH levels were significantly increased in cells treated with TNPs at doses higher than 5 μg/mL. An increased LDH production was observed in the cells treated with 40, 80, and 160 μg/mL (*p* < 0.0001). These results suggested that TNPs increased LDH levels in a dose-dependent way, which was similar to CCK8 assay.

### TNPs Exposure Promotes Inflammation

**Fig. 2:**
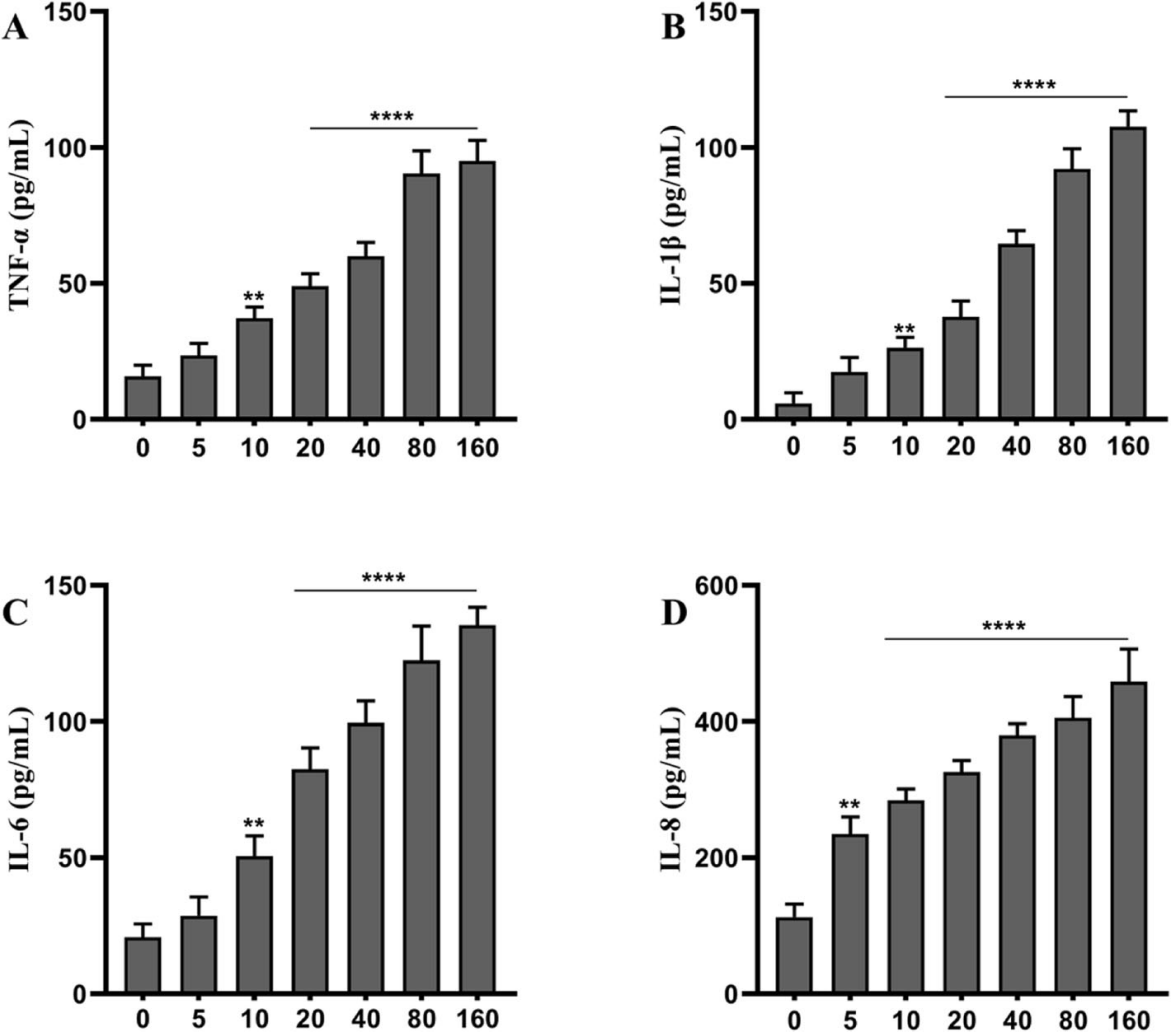
Different concentrations of TiO2-NPs (μg/mL) exposure on inflammation of SH-SY5Y cells at 72 h. (Compared with the control group, ^****^p < 0.0001)

The inflammatory response after TNPs exposure was analyzed using ELISA. After cells were treated with various TNPs concentrations for 72 h, the levels of TNF-α, IL-1β, IL-6, and IL-8 were measured. As shown in Fig. 2, IL-8 secretion was upregulated in all TNPs-treated cells; the levels of TNF-α, IL-1β, IL-6, and IL-8 were uniformly elevated in cells treated with doses higher than 5 μg/mL (*p* < 0.01). Moreover, the inflammation was significantly higher in cells treated with doses higher than 10 μg/mL group (*p* < 0.0001). Altogether, TNPs enhanced inflammation in a dose-dependent way.

Our results indicated that TNPs induced inflammatory injury in a dose-dependent way. We adopted a TNPs concentration of 80 μg/mL and an exposure period of 72 h in the following experiments to explore the role of necroptosis in the neurotoxicity of TNPs.

### Nec-1 Co-treatment Inhibits Neurotoxicity of TNPs

**Fig. 3:**
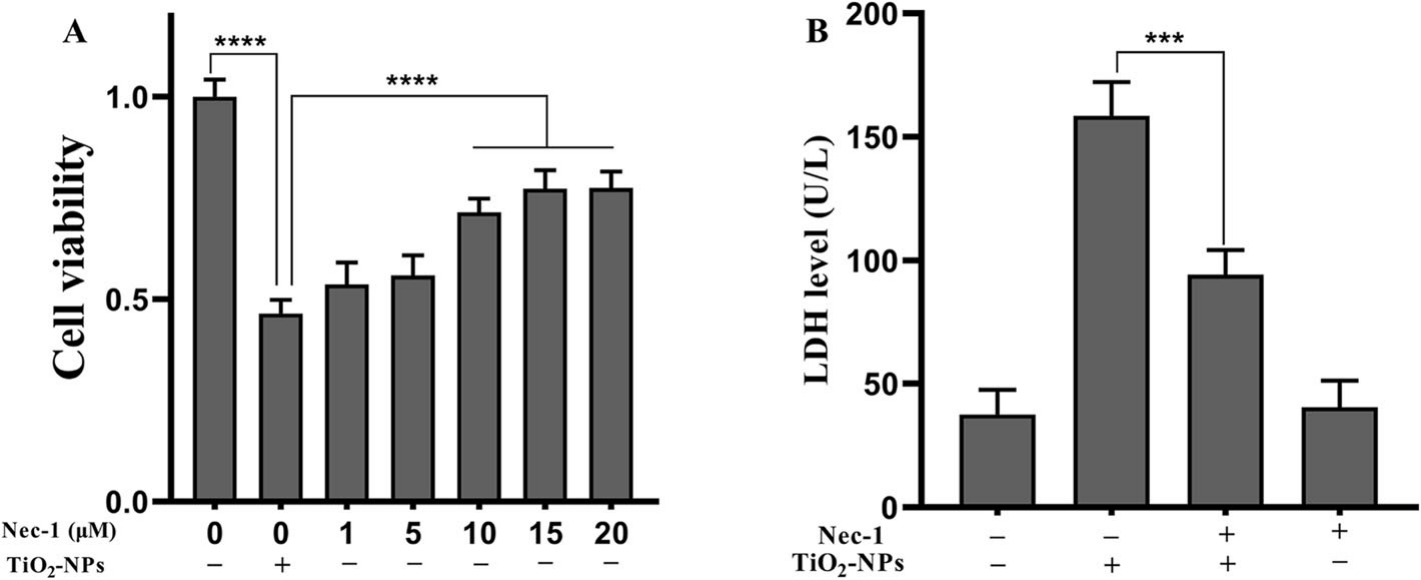
The effects of Nec-1 on cell viability and LDH after TNPs exposure. (^***^p < 0.001, ^****^p < 0.0001)

To analyze the role of necroptosis in inflammatory injury, we co-treated cells with Nec-1 (a necroptosis inhibitor) and TNPs. As shown in Fig. 3a, SH-SY5Y cells were exposed to TNPs or TNPs+Nec-1 (1, 5, 10, 15, or 20 μM), and we found that Nec-1 dramatically ameliorated the TNPs-induced reduction in cell viability (10, 15, 20 μM) (*p* < 0.0001). As cell viability in the 15 μM- and 20 μM-treated groups was not higher than that in the groups treated with 10 μM *(p* = 0.6643 and *p* = 0.6292), we co-treated cells with 10 μM Nec-1 to analyze the effect on membrane integrity.

After culturing cells with TNPs or TNPs + Nec-1, we found that LDH levels in the TNPs + Nec-1 group were much lower than that in the TNPs alone group (*p* = 0.0005). Besides, treatment with Nec-1 alone did not increase LDH leakage (*p* = 0.9878).

Conclusively, 10 μM Nec-1 could effectively inhibit the cytotoxicity of TNPs and was not toxic to cells.

**Fig. 4:**
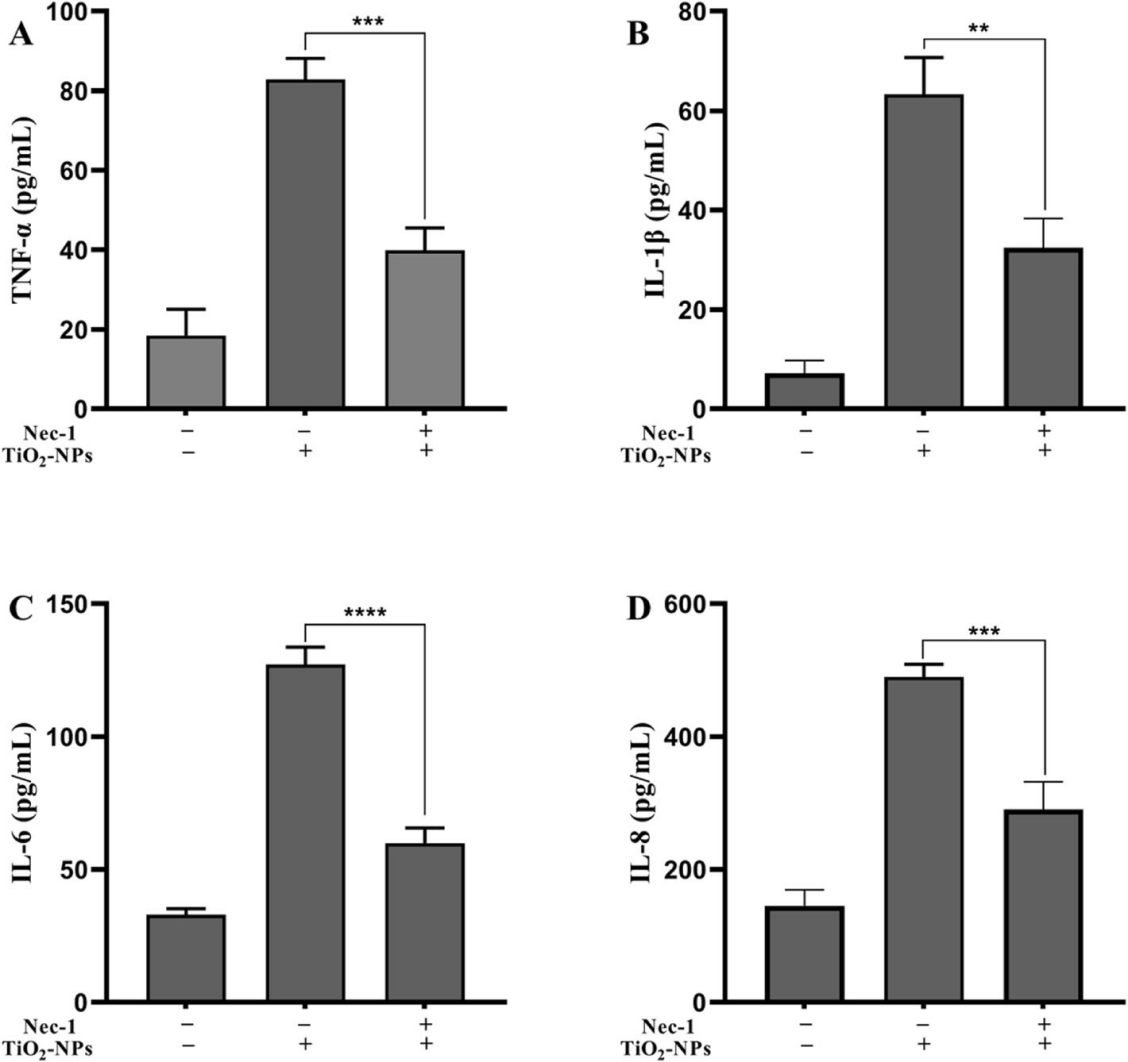
The effects of Nec-1 on inflammation after TNPs exposure. (^**^p < 0.01, ^***^p < 0.001, ^****^p < 0.0001)

To analyze the anti-inflammatory capability of Nec-1, cells were cultured with TNPs or TNPs + Nec-1. As shown in Fig. 4, the production of TNF-α (*p* = 0.003), IL-1β (*p* = 0.0013), IL-6 (*p* < 0.0001), and IL-8 (*p* = 0.0004) was significantly lower in the TNPs + Nec-1 group than that in the TNPs group.

Those results imply that Nec-1 can mitigate inflammatory responses induced by TNPs exposure.

### Silencing RIP1 Mitigates the Inflammatory Injury Induced by TNPs

**Fig. 5:**
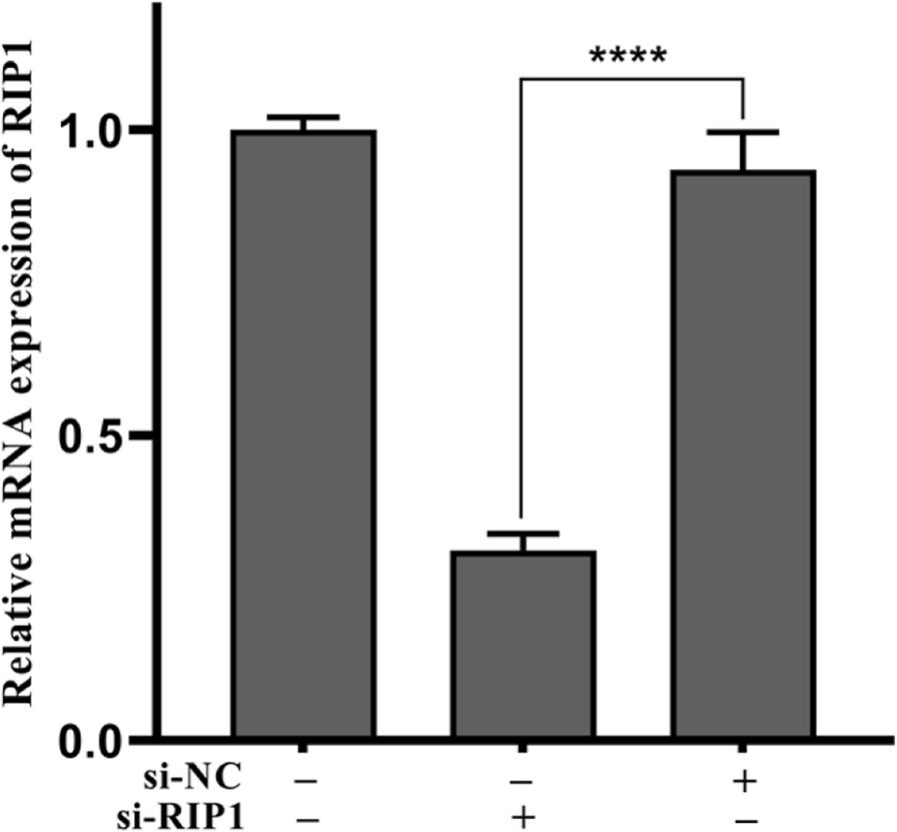
The mRNA expression after SH-SY5Y cells transfected with si-RIP1. (^****^p < 0.0001)

**Fig. 6:**
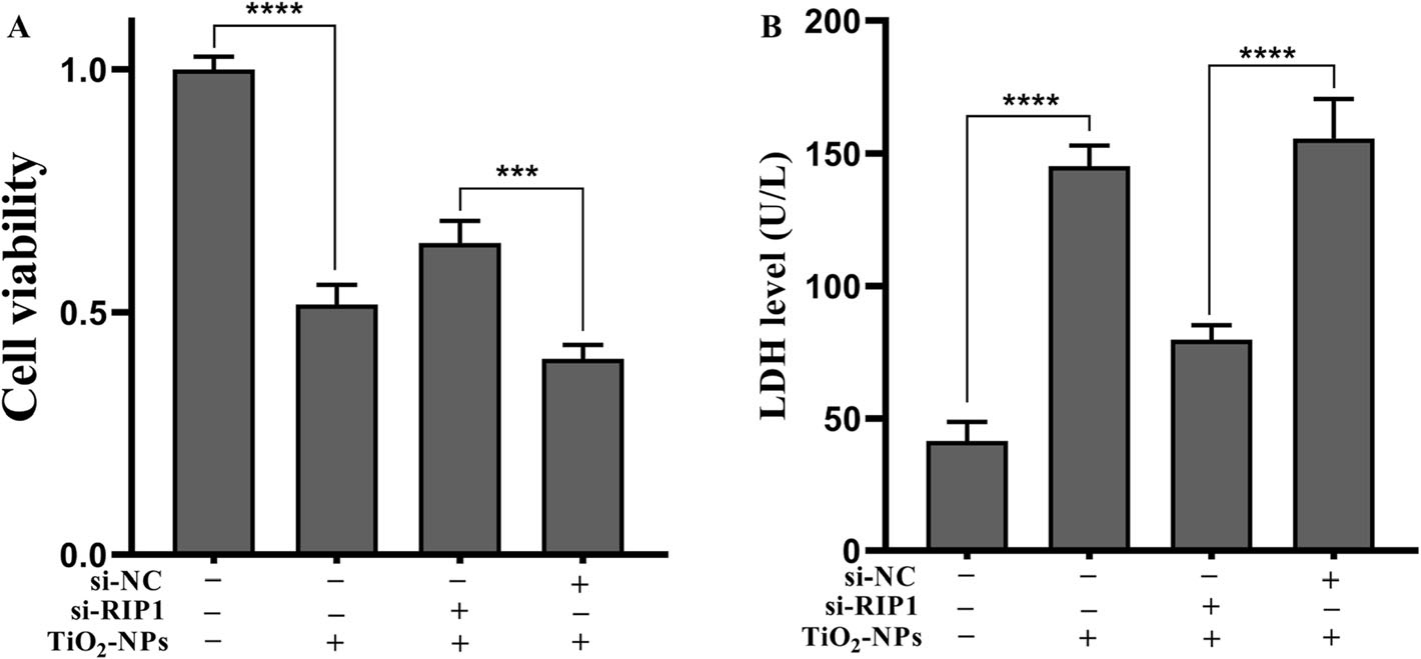
Cell viability and LDH leakage after mutant and wild-type cells were exposed to TNPs. (^***^p < 0.001, ^****^p < 0.0001)

**Fig. 7:**
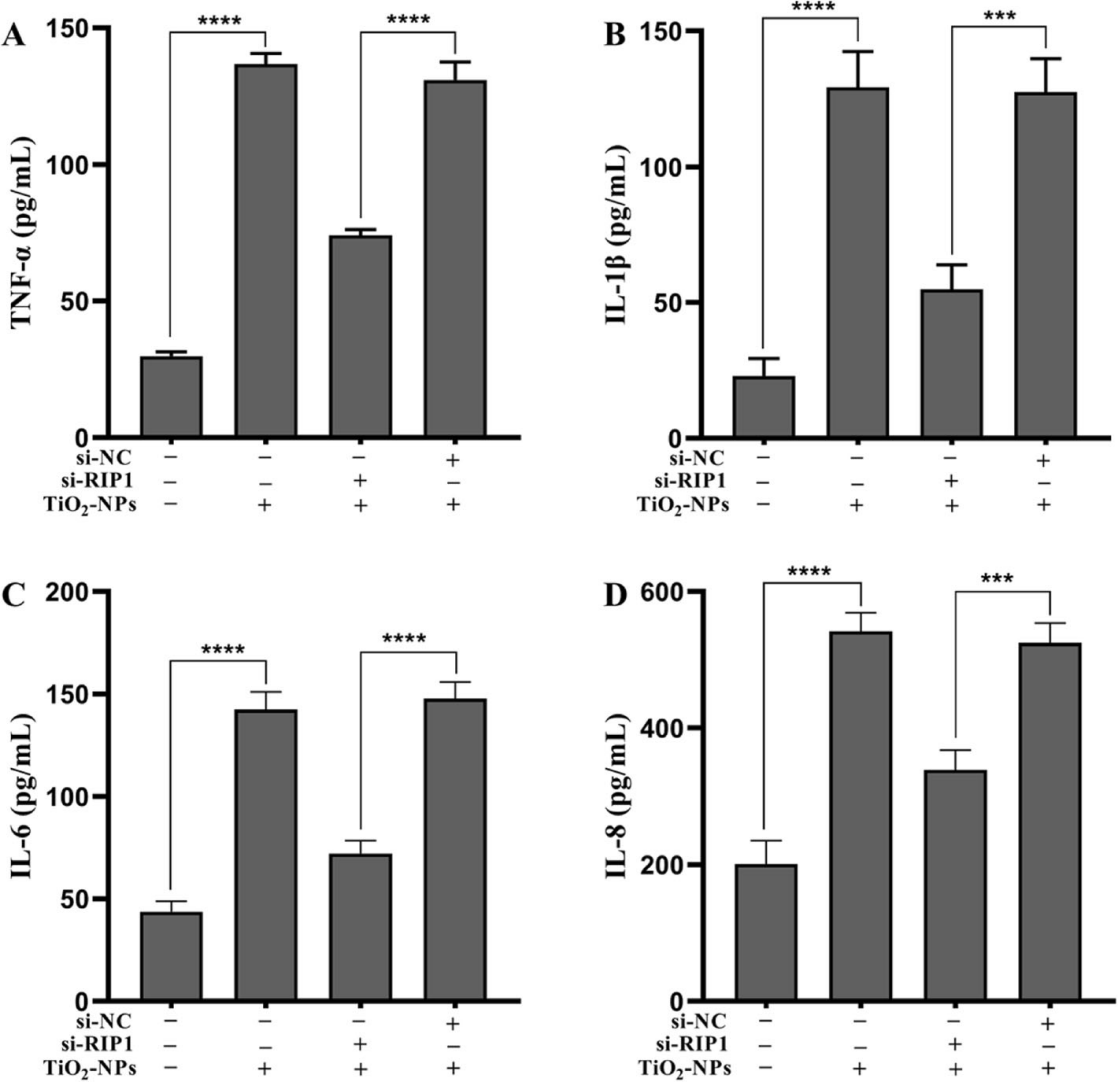
Inflammation after mutant and wild-type cells were exposed to TNPs. (^***^p < 0.001, ^****^p < 0.0001)

To determine whether Nec-1 abrogated inflammatory injury induced by TNPs through RIP1, we effectively silenced RIP1 expression of cells using siRNA (Fig. 5, *p* < 0.0001). Next, the inflammatory injury after TNPs exposure on mutant and wild-type cells was measured. Figure 6a demonstrates that cell viability in the TNPs + si-RIP1 group was remarkably higher than that in the TNPs + si-NC group (*p* = 0.0002). LDH production (Fig. 6b, *p* < 0.0001) and levels of TNF-α (*p* < 0.0001), IL-1β (*p* = 0.0001), IL-6 (*p* < 0.0001), and IL-8 (*p* = 0.0001) (Fig. 7) in the TNPs + si-RIP1 group were dramatically lower than that in the TNPs + si-NC group. These results indicated that TNPs promoted inflammatory injury through RIP1.

## Discussion

In this study, we found that TNPs exposure induced cytotoxicity on SH-SY5Y cells in a dose-dependent manner and that necroptosis was involved in the neurotoxicity of TNPs. To explore the role of necroptosis, we exposed cells to TNPs with or without Nec-1 (a potent necroptosis inhibitor) [17, 18], and measured the cell viability, LDH leakage, and the levels of inflammatory cytokines. Our data suggest that Nec-1 co-treatment can mitigate inflammatory injury induced by TNPs. Since studies have shown that Nec-1 exerts its effects by suppressing RIP1 activity, we transfected cells with si-RIP1 to determine whether RIP1 was involved in the protective effects of Nec-1. Mutant and wild-type cells were treated with TNPs, and the inflammatory injury was assessed. This indicated that cell viability in the si-RIP1 group was higher than that in the si-NC group, and levels of LDH leakage and inflammatory cytokines were lower in the si-RIP1 group than that in the si-NC group after exposure to TNPs. In conclusion, the present study revealed for the first time that necroptosis is involved in the TNPs-induced inflammatory injury on cells.

We reconfirmed the neurotoxicity of TNPs in our in vitro study. After SH-SY5Y cells were exposed to various concentrations of TNPs for various incubation times, cell viability was assessed using the CCK8 assay. As shown in Fig. 1a, TNPs exposure reduced cell viability in a dose-dependent way; after 48 h and 72 h exposure, cell viability in the 80 and 160 TNPs groups was decreased dramatically as compared with the control group (*p* < 0.001). To further confirm cytotoxicity, we also measured the LDH leakage. The data in Fig. 1b revealed that LDH production increased in a dose-dependent way after 72 h of exposure, and the LDH levels in the 40, 80, and 160 groups were remarkably higher than that in the control group (*p* < 0.0001). Because previous studies have shown that neuroinflammation can be promoted by exposure to TNPs [19], the levels of TNF-α, IL-1β, IL-6, and IL-8 were also measured. Figure 2 illustrates that the levels of those four inflammatory cytokines were significantly upregulated as compared with the control group and were dramatically higher in the groups treated with 20 to 160 μg/mL (*p* < 0.0001). Taken together, our results indicated that TNPs could induce neurotoxicity in a dose-dependent way, which is consistent with previous studies. TNPs can be absorbed by neuronal cells and inhibit proliferation [20–22]. Furthermore, TNPs exposure may decrease cell viability and promote LDH leakage in a dose-dependent manner [23–27].

We co-treated cells with Nec-1 to clarify whether necroptosis was involved in the neurotoxicity caused by TNPs. After cells were treated with indicated concentrations of TNPs with or without Nec-1, the cell viability, LDH leakage, and inflammation were assessed. Figure 3a shows that the reduction in cell viability after TNPs exposure was significantly inhibited by co-treatment with 10 μM Nec-1. Meanwhile, Fig. 3b revealed that treatment with 10 μM Nec-1 did not increase the LDH levels, and the LDH levels in cells treated with TNPs + Nec-1 group were dramatically lower than that in the TNPs alone group. Next, we measured the effects of co-treatment with Nec-1 on inflammation induced by TNPs. Figure 4 illustrates that the levels of TNF-α, IL-1β, IL-6, and IL-8 were remarkably lower in the cells treated with TNPs + Nec-1. These results reverified that Nec-1 could protect cells from death and inflammatory processes [28, 29].

Finally, since RIP1 is the target of Nec-1, we assessed whether RIP1 could regulate the neurotoxicity of TNPs. We constructed mutant cells by silencing RIP1 expression (Fig. 5). Data from Fig. 6 and Fig. 7 revealed that after TNPs exposure, the cell viability was higher, and LDH, TNF-α, IL-1β, IL-6, and IL-8 levels were lower in the si-RIP1 group than that in the si-NC, which suggests that TNPs promote cytotoxicity through RIP1. Likewise, several studies have uncovered that RIP1 can both regulate cell death and inflammatory processes [30, 31].

Although we report for the first time that Nec-1 can mitigate the neurotoxicity of TNPs by suppressing the necroptosis signaling pathway, our study has a few limitations. Firstly, the cytotoxicity of nano-sized materials differs from their bulk ones and could be largely influenced by surface properties [32], which may explain the contrary result of increased cell viability found by Sebastián et al. [33]. Therefore, it is essential to comprehensively evaluate nanotoxicity on subjects from multiple aspects, such as cell proliferation, membrane integrity, oxidative stress, inflammation, mitochondrial function, cell cycle, cytoskeleton, and epigenetics. Secondly, RIP1 can recruit RIP3 to form a functional necrosome, a vital activator of MLKL, to initiate necroptosis [34]. We hypothesize that RIP3/MLKL could be involved in the neurotoxicity of TNPs, which should be explored in future work. Furthermore, the associations of RIP3/MLKL with the neurotoxicity of TNPs should also be evaluated. Thirdly, reactive oxygen species (ROS), as the main mechanisms underlying neurotoxicity of TNPs [35, 36], were reported to be the upstream signal of RIP1 [37]. Therefore, whether ROS are the upstream of RIP1 in the neurotoxicity of TNPs should be discussed further. Fourthly, cells should be exposed to non-toxic concentrations (TNPs < 5 μg/mL) for a long-term period (more than 72 h) to assess chronic cytotoxicity. Fifthly, we should evaluate the role of necroptosis in the neurotoxicity of TNPs in more neuronal cell lines and primary human and animal neuronal cells.

## Conclusions

Our study revealed necroptosis as another programmed cell death mechanism involved in the neurotoxicity caused by TNPs. In this study, we found that TNPs could induce inflammatory injury in SH-SY5Y cells in a dose-dependent way and that Nec-1 (a potent inhibitor of necroptosis) co-treatment could ameliorate those harmful effects. RIP1, the target of Nec-1, was silenced by si-RNA, which effectively mitigated inflammatory injury induced by TNPs exposure. In conclusion, Nec-1 inhibited the inflammatory injury of SH-SY5Y cells induced by TNPs exposure by targeting the RIP1 pathway. The upstream and downstream signaling pathways of RIP1 involved in the neurotoxicity of TNPs should be assessed further.

## Materials and Methods

### TNPs Preparation and Cell Culture

We previously characterized TNPs and prepared them according to our previously published procedure [38]. Briefly, TNPs were dissolved in RPMI 1640 to various concentrations of 5, 10, 20, 40, 80, and 160 μg/mL. The TNP solution was sterilized and sonicated (300 W, 10 min) at room temperature to keep the particles from aggregating before treatment. Cells in culture medium unexposed to TNPs served as the control group. SH-SY5Y cells, bought from the Cell Bank of the Shanghai Institute of Life Sciences, Chinese Academy of Sciences, were cultured in RPMI 1640 medium (HyClone, Logan, UT, USA) supplemented with 10% fetal bovine serum (Gibco, a product line of Thermo Fisher Scientific, Waltham, MA, USA), 100 U/mL penicillin, and 100 μg/mL streptomycin at 37 °C in a humidified incubator with 5% CO_2_.

### Cell Viability and LDH Leakage Assay

Cell viability was measured using a cell counting kit-8 (CCK8. Dojindo, cat no.CK04). In brief, 1 × 10^4^ SH-SY5Y cells were placed in a 96-well plate and cultured in an incubator at 37 °C (5% CO_2_) for 24 h before exposure to TNPs. Cells were then incubated with TNPs at various concentrations (0, 1.25, 2.5, 5, 10, 20, 40, and 80 μg/mL) for another 24 h. After treatment, cells were incubated with CCK-8 for another 2 h. Next, the optical density (OD) was measured at 450 nm by placing the 96-well plate into a microplate reader (BioTek, Winooski, VT, USA). The cell viability of each group, expressed as a percentage, was calculated as (ODtreated−ODblank)/(ODcontrol − ODblank) × 100%.

Membrane integrity was measured by a commercial kit (Nanjing Jiancheng Bioengineering Institute, China). After SH-SY5Y cells were exposed to various concentrations of TNPs for 72 h, LDH secretion was analyzed according to the manufacturer’s instructions.

### Inflammatory Response

ELISA was applied to measure the levels of TNF-α, IL-1β, IL-6, and IL-8 produced by SH-SY5Y cells. Briefly, SH-SY5Y cells were exposed to TNPs for 72 h, and the supernatant was collected for analysis as per manufacturer’s instructions (Elabscience Biotechnology Co., Ltd.).

### siRNA Transfection

Following the manufacturer’s protocol, 100 nM concentration of si-RIP1 or negative control siRNA (si-NC) was transfected into cells by using Lipofectamine 2000. The efficiency of gene silencing by siRNA was estimated with real-time PCR.

### Real-Time PCR

RNA of cells exposed to various concentrations of TNPs was isolated using the RNAqueous kit (Ambion Inc., Austin, TX, USA) based on the manufacturer’s instructions. The relative expression of RIP1 was measured by real-time PCR. The relative mRNA levels of RIP1 were normalized to β-actin expressions.

### Statistical Analysis

SPSS 11.0 software (SPSS Inc., Chicago, IL, USA) was used to analyze data. One-way analysis of variance was used to compare groups’ means. *p* < 0.05 was considered statistically significant.

## Data Availability

Available from the manuscript.

## Abbreviations

TNPs: Titanium dioxide nanoparticles
Nec-1: Necrostatin-1
RIP: Receptor interacting protein kinase
MLKL: Mixed lineage kinase domain-like protein
ROS: Reactive oxygen species
